# Therapeutic Use of G4-Ligands in Cancer: State-of-the-Art and Future Perspectives

**DOI:** 10.3390/ph17060771

**Published:** 2024-06-13

**Authors:** Sara Iachettini, Annamaria Biroccio, Pasquale Zizza

**Affiliations:** Translational Oncology Research Unit, IRCCS—Regina Elena National Cancer Institute, Via Elio Chianesi, 53, 00144 Roma, Italy; sara.iachettini@ifo.it (S.I.); annamaria.biroccio@ifo.it (A.B.)

**Keywords:** G-quadruplex, G4-ligands, Cancer therapy, Nucleic acids

## Abstract

G-quadruplexes (G4s) are guanine-rich non-canonical secondary structures of nucleic acids that were identified in vitro almost half a century ago. Starting from the early 1980s, these structures were also observed in eukaryotic cells, first at the telomeric level and later in regulatory regions of cancer-related genes, in regulatory RNAs and within specific cell compartments such as lysosomes, mitochondria, and ribosomes. Because of the involvement of these structures in a large number of biological processes and in the pathogenesis of several diseases, including cancer, the interest in G4 targeting has exponentially increased in the last few years, and a great number of novel G4 ligands have been developed. Notably, G4 ligands represent a large family of heterogeneous molecules that can exert their functions by recognizing, binding, and stabilizing G4 structures in multiple ways. Regarding anti-cancer activity, the efficacy of G4 ligands was originally attributed to the capability of these molecules to inhibit the activity of telomerase, an enzyme that elongates telomeres and promotes endless replication in cancer cells. Thereafter, novel mechanisms through which G4 ligands exert their antitumoral activities have been defined, including the induction of DNA damage, control of gene expression, and regulation of metabolic pathways, among others. Here, we provided a perspective on the structure and function of G4 ligands with particular emphasis on their potential role as antitumoral agents. In particular, we critically examined the problems associated with the clinical translation of these molecules, trying to highlight the main aspects that should be taken into account during the phases of drug design and development. Indeed, taking advantage of the successes and failures, and the more recent technological progresses in the field, it would be possible to hypothesize the development of these molecules in the future that would represent a valid option for those cancers still missing effective therapies.

## 1. G4s Are Non-Canonical Secondary Structures Spontaneously Originating from G-Rich Sequences of Nucleic Acids

Deoxyribonucleic acid (DNA) and ribonucleic acid (RNA) are key players in the continuity of life. Indeed, these macromolecules, carriers of genetic information, are able to finely regulate the behavior of cells and, consequently, the features of the entire organism.

In the early 1950s, James Watson and Francis Crick, upon collecting and analyzing some existing piece of data relative to the molecular structure of DNA, formulated the so-called double helix theory, according to which two DNA strands, held together by hydrogen bonds between the bases on opposite strands, assume a characteristic twisted ladder shape [1]. 

**Figure 1:**
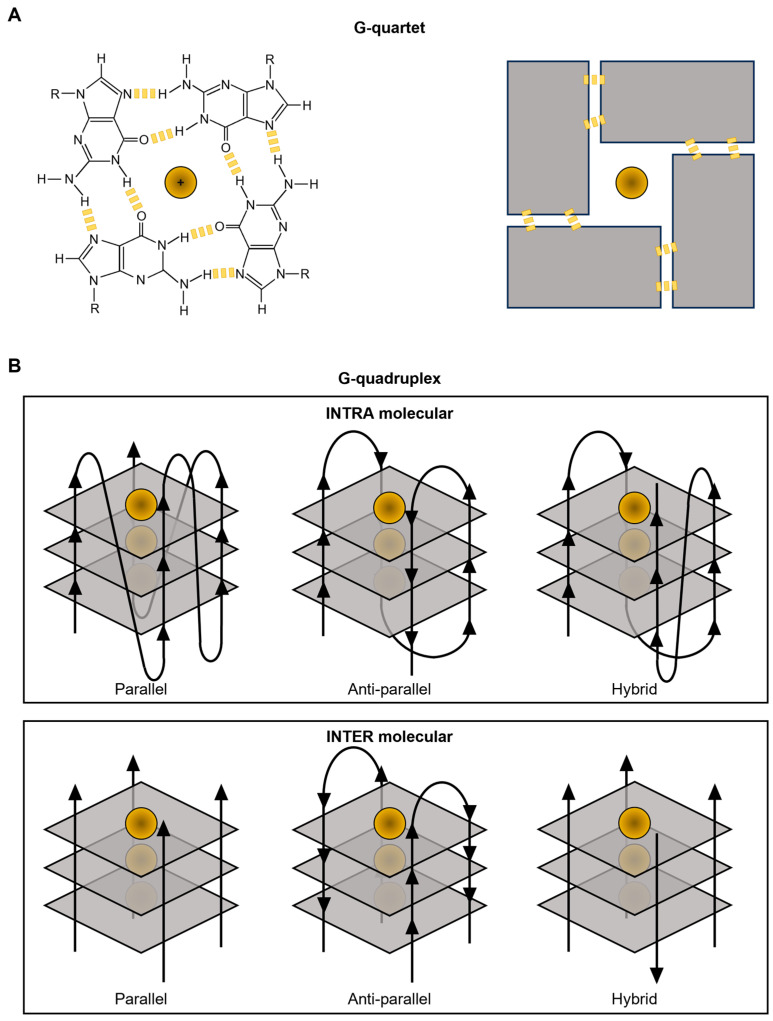
G-quadruplex structure. Schematic representation of G-quartet (**A**) and G-quadruplex structures (**B**).

Despite the fact that the double helix (also known as B-DNA) was long believed to only have the three-dimensional conformation assumed by DNA under folding conditions, more than 20 different kinds of alternative secondary structures with potential regulatory functions have been described over time [2]. This is the case of the G-quadruplexes (G4s) for example, four-stranded structures arising from the guanine (G)-rich sequences of DNA and RNA (Figure 1). At the structural level, the building blocks of G4s are represented by the so-called G-quartets, planar structures originating from the association of four guanines into a cyclic arrangement that is stabilized by Hoogsten-type hydrogen bonding (Figure 1A). For the assembly of the G4s, G-quartets stack on top of one another, forming four-stranded helical structures that are finally stabilized by the presence of monovalent cations, usually Na^+^ or K^+^, localized at the center of the different tetrads, and coordinated to the O6 of the guanines [3]. G4 structures can either originate from a single nucleic acid strand (intramolecular structures) or be the result of the interaction among multiple molecules (intermolecular structures) (Figure 1B). Finally, these structures can adopt multiple topological conformations that, depending on the length and composition of the nucleic acids, pattern of strand polarities, and orientation of connecting loops, can be distinguished into parallel, antiparallel, and hybrid forms (Figure 1B).

Despite the first description of G4 structures dating back to about 50 years ago, these structures were long considered to have been artifacts originating under particular experimental conditions in vitro. Only in the last few years, thanks to technological advancements and taking advantage of structure-specific antibodies (e.g., BG4, an antibody that is able to recognize G4 structures in cells) [4] and/or G4 ligands (i.e., molecular probes with G4-binding activity) [5], real evidence of the existence of these non-B structures has been definitively obtained. In this context, a particular relevance has been assumed by single-molecule fluorescence microscopy and atomic force microscopy (AFM), two techniques that, through real-time visualization of G4 in cells, have provided insights into the dynamics and spatial organization of these structures in vivo [6,7].

Of note, cell-based analyses described G4s as structures originating at telomeres, repetitive G-rich DNA regions localized at chromosome ends [8]. At the biological level, telomeres have a key role in buffering chromosome erosion, a process arising from the inability of DNA polymerases to replicate the terminal portion of linear chromosomes (an end-replication problem) which is potentially detrimental to genetic information. In somatic cells, excessive telomere shortening induces senescence, a defensive process inhibiting cell proliferation. Conversely, stem and germ-line cells counteract the phenomenon of telomere erosion through the activity of telomerase, an enzyme that, by re-elongating telomeres, is able to confer the endless replicative potential typical of these cells [9]. Similarly to stem and germ-line cells, several tumors have also been found to acquire their immortalized phenotype through the capability of re-activating the expression of this enzyme [10]. In this context, the therapeutic advantages that could be derived from their pharmacological stabilization have quickly attracted scientists, especially those involved in cancer research [11].

More recently, G4 structures have also been identified outside telomeres, in G-rich chromatin regions. In particular, bioinformatic analyses performed by applying algorithms for the identification of G4-forming motifs detected several thousands of sequences able to potentially fold into G4 structures, several of which were localized within regulatory regions of cancer-related genes (e.g., KRAS, MYC, KIT, VEGF, VEGF-R, mTOR, HIF1a) [12,13]. In particular, these extra-telomeric G4s have been found within gene promoters, in enhancer regions, and in the junctions between introns and exons, indicating that these structures might play an active role in gene regulation. Moreover, additional G4-forming sequences have been recently found within non-coding RNAs [14], in the untranslated regions (UTRs) of certain mRNAs [15], and within sequences of mitochondrial [16] and ribosomal DNA [17,18]. Notably, the effective capability of the identified sequences to form G4s in cells has been demonstrated, case by case, through the application of analytical techniques such as chromatin immunoprecipitation (ChIP) assays, which were performed with antibodies directed against the G4 structures. More recently, through the introduction of the ChIP-seq, an analytical tool developed by coupling ChIP assays with high-throughput sequencing approaches, it has been possible to map the G4 structures at a genome-wide level [19]. Interestingly, the identification of these extra-telomeric G4s within the regulatory regions of the human genome has contributed to renewing and reinforcing the interest of scientists in these structures and has paved the way to a new phase in the development of the G4-targeting molecules.

## 2. Therapeutic Relevance of G4 Structures in Cancer

**Figure 2:**
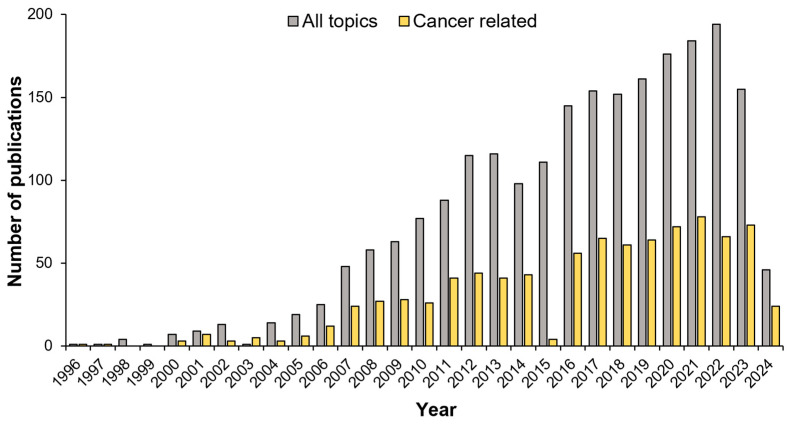
Timeline of G-quadruplex ligands publications. Timeline of publications about G-quadruplex ligands. The grey bars indicate the total amount of publications about G-quadruplex ligands, the yellow bars indicate the number of publications related to cancer. The results, derived from PubMed (https://pubmed.ncbi.nlm.nih.gov), are expressed as the number of published papers per year.

Over the last few years, there has been an exponential growth in the number of studies proposing G4 ligands as antitumoral drugs (Figure 2). 

**Figure 3:**
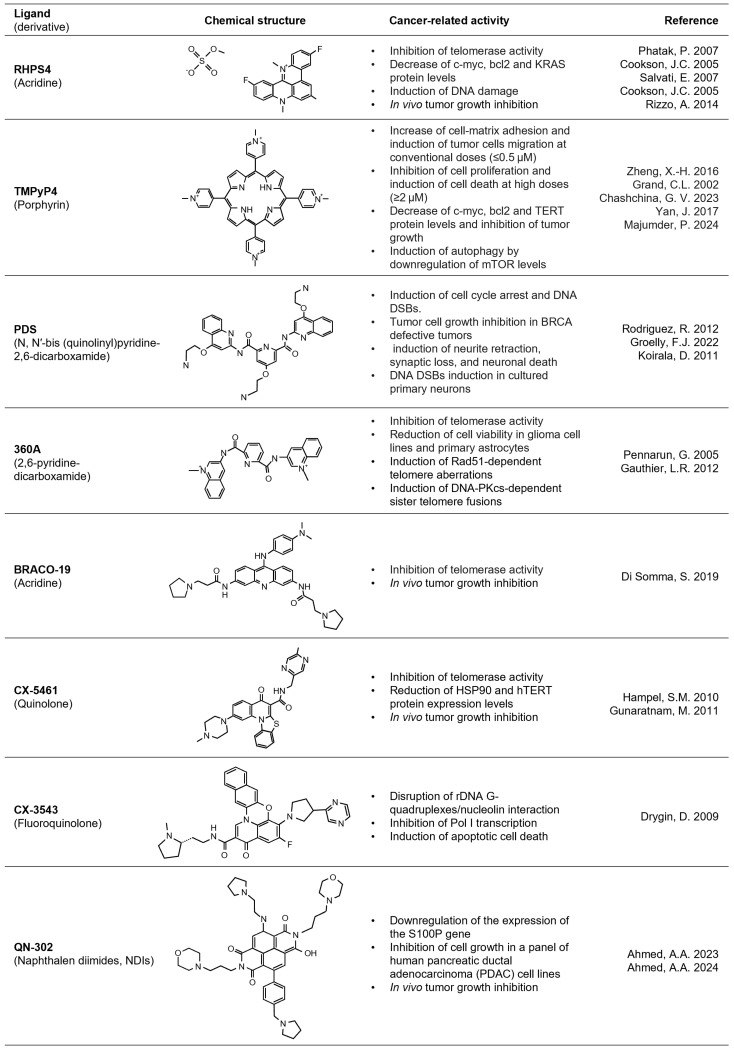
G-quadruplex ligands. List of representative G4-interacting ligands mentioned in the text. Each G4 ligand was drawn according to chemical structures available in the PubChem database (https://pubchem.ncbi.nlm.nih.gov).

In detail, the development of these novel therapeutic strategies was originally driven by the idea that these molecules, stabilizing G4 structures at telomeres, were able to physically inhibit telomerase activity, consequently impairing the endless replication of cancer cells [11]. However, studies aimed at evaluating the effect of the G4 ligands in cancer cells evidenced short-time antitumoral effects, not compatible with telomerase inhibition, a process that would take a long time and several cell-division events before inducing cell death [14]. In this context, a very exemplificative case is that of pentacyclic acridine 3,11-difluoro-6,8,13-trimethyl-8H-quino[4,3,2-kl]acridinium methosulfate (RHPS4, Figure 3), where a G4 ligand was initially proposed as a telomerase inhibitor and was then demonstrated to also promote its antitumoral activity in telomerase-negative (alternative length telomere; ALT) tumor cells [20]. Further studies aimed at investigating in-depth the telomerase-independent mechanism(s) of RHPS4 activity demonstrated that this drug promotes telomere dysfunctions, topological stress, and the accumulation of DNA damage [11], all events that justify the short-time antitumoral response observed in cancer cells exposed to G4 ligands [13].

More recently, the antitumoral potential of G4 ligands has been demonstrated to also proceed through their capability to affect gene expression, a phenomenon that is associated with the stabilization of G4 structures localized within the regulatory elements of cancer-related genes. In this context, a particularly relevant contribution was provided by the studies of Hurley, whose group demonstrated the efficacy of TMPyP4, a small cationic porphyrin ligand, to impair c-Myc expression through the stabilization of a G4 within its promoter [21]. Since then, different structural classes of G4 ligands, such as Pyridostatin (PDS), 360A, BRACO-19, and CX-5461 (Figure 3), have been demonstrated to exert a suppressive activity towards c-Myc expression. Similar to c-Myc, several other proto-oncogenes, such as Bcl-2, c-Kit and RET, have been demonstrated to form parallel G4 structures within their promoters and, as such, can be targeted by G4 ligands [22]. Furthermore, RHPS4, already cited for its telomere-related activities, has been found to inhibit the expression of the vascular endothelial growth factor receptor (VEGF-R). At the functional level, the alteration of VEGF-R promotes an impairment of vascularization and, consequently, an inhibition of tumor growth and dissemination. Again, a recent study published by Majumder and colleagues demonstrated the capability of TMPyP4 to impair the expression of mTOR, a key factor in the autophagic process [23]. 

During this time, the therapeutic potential of G4s has extended beyond the regulation of gene expression. For example, CX-5461 was found to promote synthetic lethality in tumors defective for homologous recombination process [24,25], while PDS was demonstrated to trap topoisomerase II on DNA [26]. Moreover, it was demonstrated that both these ligands are able to trigger TOP2A-mediated DNA double-stranded breaks via G4 stabilization [27].

Altogether, these findings underline the therapeutic relevance of G4 structures in the oncologic field and represent a significant boost to promoting the development of novel and effective ligands that are applicable to patients in the clinical setting.

## 3. Limitations of G4-Based Antitumoral Therapies

As anticipated, the possibility of targeting cancer cells through the pharmacological stabilization of naturally occurring G4 structures has long been considered by chemists, physicists, biologists, and clinicians as an opportunity for developing molecules that are able to selectively counteract tumor formation and progression. In agreement with this, the number of G4 ligands has significantly grown over the last 25 years, constituting a superfamily of molecules, with up to 3000 different members to date, distinguished for their structure and biological activity (see Figure 3 for notable examples reported in the text) [21,23,28,29,30,31,32,33,34,35,36,37,38,39,40,41,42,43,44,45,46]. 

Despite the therapeutic potential demonstrated by several of these molecules, few G4 ligands have entered pre-clinical evaluation or the early phases of clinical trials, and none of them have been entered into clinical practice so far. Notably, the limited success of G4 ligands is due, in part, to the fact that a number of these molecules, despite their effectiveness at the biochemical and biophysical levels, show poor activity and/or selectivity when translated into cells.

For example, TMPyP4, initially reported as an exceptional G4 ligand, was then found to recognize both duplex and triplex DNA structures to the same extent [47]. Additionally, certain G4-stabilizing agents, even though they had shown excellent activity in cell-based assays, were then demonstrated to have poor effectiveness or were found to induce side- and/or off-target effects when translated into preclinical experimental models. A typical example, in this context, is represented by RHPS4 which, despite its marked antitumoral potential, demonstrated severe cardiotoxic effects when tested in guinea pig models [32]. Another example is BRACO-19, a G4 ligand that, despite showing potent activity against telomerase in cell-free assays, manifested several biopharmaceutical limitations when tested in vivo. In particular, the poor membrane permeability of this 3,6,9-trisubstituted acridine derivative, together with its sub-optimal pharmacological profile, have been found to dramatically hamper its bioavailability and therapeutic effectiveness [48]. Finally, due to a deep imbalance between the rapid development of novel G4-stabilizing agents and the complexity, timing, and high cost of the translational studies, several potentially promising molecules are often neglected and not sufficiently investigated.

The result of this very complex and diverse situation is that only a few molecules have entered clinical trials to date. Among these, CX-3543, also known as quarfloxin, is a fluoroquinolone originally designed to target the G4 structure localized within the promoter of c-MYC [49]. However, this molecule has been experimentally demonstrated to also stabilize G4 structures originating in the ribosomal DNA, promoting robust inhibition of ribosome biogenesis that finally results in a general impairment of gene transcription [44]. Despite a number of promising results, clinical evaluation of this molecule was interrupted due to the high binding affinity, that emerged during the phase III trials, of this molecule with albumin [50]. Another fluoroquinolone with marked G4-stabilizing activity is the CX-5461, or Pidnaruex. This molecule, which is a structural analog of CX-3543, is able to recognize, bind, and stabilize a broad spectrum of G4 structures within the genome, including those originating at the telomeres and in the promoter of the oncogenes c-MYC and c-KIT [51]. In addition, CX-5461 has been demonstrated to impair ribosomal RNA synthesis by inhibiting the binding of the SL1 pre-initiation complex and the RNA polymerase I complex to the ribosomal DNA [52]. Of note, based on the preclinical results showing the capability of CX-5461 to trigger synthetic lethality in BRCA-deficient tumors resistant to PARP inhibitors [24,53], this G4 ligand is currently entered into phase Ib clinical trials (NCT04890613) as a drug for the treatment of cancer patients with germline mutations of BCRA1/2 and/or alterations in the genes encoding for non-homologous end joining factors such as LIG4 and DNA-PK [24]. Another very promising G4 ligand is QN-302 (alias: SOP1812), a recently developed naphthalene diimide (NDI) derivative, characterized by the presence of 4 phenyl substituents directly attached to an NDI core [54]. Notably, QN-302 has been designed to selectively recognize, bind, and stabilize parallel G4 structures, a feature conferring to this ligand the specificity for G4 structures localized outside the telomeres. Of note, preclinical studies performed to characterize the biological behavior of QN-302 have evidenced that this molecule shows excellent bioavailability and a G4-stabilizing activity largely superior to other NDI-derivatives [54]. At the mechanistic level, the antitumoral properties of QN-302 are mainly attributable to the capability of this molecule to bind a G4 structure located within the promoter of S100P and to inhibit the expression of this factor that is known to play a key regulator role in tumor proliferation and motility [45]. Based on its pharmacological properties and taking into account the promising results obtained both in vitro and in vivo, QN-302 has rapidly entered into phase Ia clinical evaluation as a potential therapeutic agent for the treatment of patients with advanced or metastatic solid tumors such as pancreatic ductal adenocarcinoma, sarcomas, gastrointestinal stromal tumors, and prostate cancer (NCT06086522) [45]. Since the enrollment of patients into this trial just started a few months ago, it will be necessary to wait some time to know the future developments and understand if this molecule effectively represents the first G4 ligand to move from the bench to the bedside.

Of note, independent of the successes and failures, the data collected so far have allowed us to understand the therapeutic potential and limitations of G4 ligands, as well as the intrinsic advantages and disadvantages of specific developmental approaches and/or analytical tools. Based on this knowledge, it will be possible to develop new ligands that may be synthesized ex novo or derived from already existing molecules which might manifest an effective curative potential towards malignancies still missing therapy. 

## 4. Optimization of G4 Ligands for Therapeutic Use in Cancer: Novel Strategies and Future Perspectives

As previously evidenced, many G4 ligands interact with G4 structures in a relatively indiscriminate manner, meaning that these molecules can recognize structures of different topologies, so exerting their activity as multi-targeting agents. Because G4s are present in both normal and cancer cells, the poor selectivity of G4 ligands represents one of the major limitations in the clinical progression of these molecules as anticancer agents. Based on this it would be necessary to modify the rationale underlying the design of G4 ligands in order to obtain molecules that are able to target the cancer cells more selectively. A possible strategy to obtain this goal is by developing ligands that recognize just one or a few G4 structures specifically associated with cancer-related functions. In view of this, the G4 ligands should be designed by taking into account the characteristics of their targets, such as the structural conformation and/or the spatial organization [55]. However, topological similarities in the skeleton of diverse G4s have dramatically limited the possibility of obtaining specificity toward individual G4 structures. Recently, high-resolution structural analyses of G4s complexed with small molecules have unveiled that loops and grooves can represent distinctive G4 elements that can be exploited for selective recognition by specific ligands [56]. In this context, an attractive strategy to enhance selectivity is represented by the possibility of targeting multimeric-G4s, two or more consecutive structures that, while introducing structural complexity to the basic G4 scaffold, can be selectively targeted [31,57]. Recently, some unconventional G4 structures have been described within the human genome. Among these, hairpin G4, structures characterized by long hairpin-forming loops, offer an extremely valuable target for the development of specific and more selective ligands [58]. Very recently, novel strategies to gain selectivity to G4 ligands have started to be explored. An example is represented by the conjugation of the G4 ligands with oligonucleotides or peptide nucleic acid (PNA) that can act as carriers and drive these molecules toward specific targets [59,60]. The idea behind the development of highly selective G4 ligands is that these molecules, through interacting with specific targets, would limit the risk of side- and/or off-target effects.

Beyond the different solutions so far discussed, a valid alternative to the enhancement of selectivity G4 ligands for specific targets is the development of molecules designed to selectively recognize cancer cells. Notably, this developmental approach employs nanoparticles or liposomes that, by interacting with cancer cells, would promote the proper delivery of the G4 ligands, limiting the risk of side and/or off-target effects [61,62].

Another key feature to take into account during the processes of design and development of novel G4 ligands is the evaluation of their pharmacokinetic properties. Indeed, it is not unusual that molecules, effective in cell-free experiments, would be poorly active in cells and/or in vivo. In view of this, the acquisition of knowledge concerning the kinetic of absorption, distribution, metabolism, and excretion of the investigated molecules would allow for the optimization of the treatment protocols.

Finally, to accelerate the clinical development of G4 ligands, it would be useful to evaluate the efficacy of the therapeutic strategies derived from the combination of these molecules with other antitumoral agents (ranging from canonical chemotherapeutic drugs up to the most recent immunomodulatory therapies). These combinatorial approaches would allow us to scale down the concentration of G4 ligands, limiting the risk of toxic effects while avoiding the acquisition of resistance by cancer cells.

Finally, an additional aspect that should be addressed concerns the validation process of G4 ligands. Indeed, looking at the most recent literature data, it is possible to note that a huge number of novel molecules are synthesized every year, and it is not rare to observe works in which dozens of G4 ligands, derived from the same lead compound and showing very similar properties in terms of mechanism of action and effectiveness, are proposed. Due to the existing differences, in terms of time and cost, between biochemical/biophysical analyses and biological assays, it is not reasonable to proceed with validating such an elevated number of new ligands. In this context, it is necessary—at the chemical level—to increase the stringency of the criteria that regulate the development of novel G4 ligands and—from a biological point of view—to develop advanced experimental models (e.g., spheroids and/or organoids) [63,64]. These tools—representing the bridge between simple cell-based experiments and very complex in vivo analyses—would speed up, reduce the costs, and facilitate the biological characterization process and the pharmacological optimization of the tested molecules [65], facilitating their access to clinical trials. 

## 5. Conclusive Remarks

In this work, we discussed the main aspects relative to the biology of G4 structures and their ligands. Our work does not have the ambition of being an extensive review of the literature but rather, it is aimed at sensitizing the readers towards an exciting research field with huge potential in cancer therapy. Considering the above-mentioned information, we have presented an overview of certain well-known G4 ligands and their antitumoral activities, focusing on their biological effects and discussing, in a critical way, their applications and corresponding limitations.

As evidenced within the manuscript, few G4 ligands with suitable pharmacokinetic properties have been discovered to date and still fewer molecules have entered clinical trials. Based on our analyses, it is reasonable to conclude that the aspects underlying the limited success of these molecules rely, first of all, on certain shortcomings in the strategies driving ligand evaluation and, secondly, on the poor target selectivity shown by some of these molecules. In view of this, it would be desirable and very useful to revisit the current manner of thinking about G4-ligands development, a process that, in our opinion, needs to essentially go through the establishment of interdisciplinary collaborations aimed at characterizing, from the beginning and at multiple levels, the newly synthesized molecules.

Notably, optimization of the drug developmental process may soon reasonably lead in enhancing the success rate of the future G4 ligands, definitively paving the way for the therapeutic use of these molecules in a number of important pathologies, the first being cancer.

## Data Availability

Not applicable.
